# Health-related quality of life and prospective caries development

**DOI:** 10.1186/s12903-016-0166-3

**Published:** 2016-02-09

**Authors:** Marie-Louise Åkesson, Elisabeth Wärnberg Gerdin, Ulf Söderström, Bernt Lindahl, Ingegerd Johansson

**Affiliations:** Department of Odontology, Section of Cariology, Umeå University, Umeå, Sweden; Dental Research Department, Public Dental Service, Region Örebro County, Örebro Sweden and Faculty of Medicine and Health, School of Health and Medical Sciences, Örebro University, Örebro, Sweden; Department of Public Dental Service, County council of Västerbotten, Umeå, Sweden; Department of Public Health and Clinical Medicine, Occupational and Environmental Medicine, Umeå University, Umeå, Sweden

**Keywords:** Dental caries, Health related quality of life, SF-36

## Abstract

**Background:**

The present study was conducted to prospectively assess the association between health-related quality of life (HRQoL) and the development of dental caries in adults in northern Sweden. The SF-36 questionnaire was used to estimate HRQoL.

**Methods:**

Adults who had (i) participated in a population-based health screening in northern Sweden between 2003 and 2009 and had completed the SF-36 questionnaire, and (ii) received a dental check-up within 1 year (*n* = 15,615) were included in the study. Of these, 9,838 had a second caries examination 2–7 years after the baseline recording. Information regarding SF-36, lifestyle factors and medical conditions was retrieved by questionnaires, and anthropometric status and blood lipid levels were measured. The association between dental caries (outcome) and SF-36 scores (exposure) with the inclusion of potential confounders was analysed by linear and logistic regression.

**Results:**

Caries increment increased significantly with decreasing scores for both physical and mental dimensions of SF-36 in women, but no association was seen in men. However, lifelong caries experience (DMFS) increased linearly with decreasing physical HRQoL in both men and women; this was also observed for the single dimension of mental HRQoL. The crude odds ratio for being in the highest caries quintile compared to the lowest when having the poorest physical HRQoL compared with the best physical HRQoL was 1.88 (95 % CI: 1.54–2.3). Several factors were identified as potential confounders in the associations between DMFS and SF-36 scores, including education level, smoking, age, medications, higher levels of total cholesterol, triglycerides, systolic blood pressure, body mass index and sugar intake. Except for education level and smoking, the effect sizes for the association between gradually decreasing SF-36 scores and increasing caries were generally moderate.

**Conclusions:**

Increased development of caries was associated with low physical HRQoL and some aspects of mental HRQoL. The mechanisms underlying these associations, which are likely confounded by both biological and lifestyle factors, remain to be elucidated. The study implies that, when possible, subjects with poor HRQoL would benefit from caries prevention measures meeting the underlying situation.

**Electronic supplementary material:**

The online version of this article (doi:10.1186/s12903-016-0166-3) contains supplementary material, which is available to authorized users.

## Background

Health status is one cornerstone of an individual’s quality of life (QoL). QoL is defined as an individual’s perception of his or her position in life in the context of the culture and value systems in which he or she lives and in relation to his or her goals, expectations, standards and concerns [1]. Health is defined as a state of complete physical, mental and social wellbeing and not merely the absence of disease or infirmity [2]. These two definitions emphasise that both QoL and health related quality of life (HRQoL) are influenced by a complex interplay of social and biological determinants [3, 4].

Dental caries is a multifactorial disease in which host resistance and susceptibility combined with lifestyle factors modify the overall outcome. The determinants of caries are complex and involve both biological and behavioural factors, such as diseases and medications, hyposalivation and impaired saliva defence functions, bacterial dysbiosis, oral muscle activity, diet, oral hygiene, education level, smoking and socioeconomic status [5].

The impact of dental caries on oral health-related quality of life (OHRQoL) has been described in a number of studies in children, adolescents and adults [6–9]. Some studies have also described this for HRQoL [10, 11], with worse scores in those with poorer dental status. It is unclear whether the OHRQoL and HRQoL scores represent independent entities and different sets of underpinning aspects in relation to dental caries. For instance, Mello dos Santos et al. [10] found no correlation between OHRQoL and HRQoL scores, whereas Broder et al. [11] found moderate negative correlations between some aspects of these scores. It may be assumed that poor dental status may have a causal effect on OHRQoL and not vice versa, whereas a bilateral relation may hide behind the association between dental caries and HRQoL, *i.e.,* poor dental status may impair life quality, but non-dental determinants for HRQoL may also be associated with an increased risk of developing caries. Examples of such potential confounding (or common soil) aspects are diseases and medications, but detailed knowledge on such factors is limited.

The instruments used to assess HRQoL are either generic or are designed to target specific conditions or parts of the body, such as the mouth. In contrast to target-specific instruments, the generic forms allow for comparisons between population groups with different diseases and medical problems [12]. The 36-question short form instrument (SF-36) is a generic instrument for measuring HRQoL that has been used in several clinical studies [13, 14]. SF-36 measures physical and mental HRQoL; it is a valid instrument for assessing HRQoL during illness and after treatment, and for comparing HRQoL in different groups [15]. The 36 questions in the SF-36 instrument cover eight dimensions of function and well-being [16]; gender- and age-specific reference data on these eight SF-36 dimensions have been described for various populations, including the Swedish population [13, 17]. In addition, two summary scores can be calculated on the basis of weighting of the individual SF-36 dimensions: the Physical Component Summary (PCS) and the Mental Component Summary (MCS) scores [18].

The aims of the present study were to evaluate the association between HRQoL and prospective dental caries status and to search for factors associated with HRQoL and dental caries. The study was nested within the population-based Västerbotten Intervention Programme (VIP) conducted in northern Sweden. The SF-36 instrument was used to estimate physical and mental HRQoL. We hypothesised that lower physical and mental HRQoL would be associated with an increased risk of developing dental caries in both men and women.

## Methods

### Study population

The participants in the present study were included in the VIP, which is a community intervention project that invites the inhabitants of Västerbotten to a health check-up at their primary health care centre the years they turn 40, 50 and 60 years of age (for a period, 30-year-olds were also entitled to a check-up at individual centres). At the health check-up, participants answer a questionnaire on health and lifestyle aspects and they are subject to a medical screening. The participation rate has varied over time, but has been approximately 66 % in recent years [19]. Furthermore, no systematic differences have been found between those who have participated and those who have declined [20]. For the present study, subjects who had participated in a VIP screening between 2003 (when the SF-36 instrument was included in the questionnaire) and 2009 (*n* = 47,341 unique subjects) and had their regular dental care provided by the Public Dental Service were eligible. Subjects with a full dental examination within a year after the VIP screening (*n* = 17,882 unique subjects) were included. After merging the VIP questionnaire data, 15,615 unique subjects fulfilled the criteria of (i) having a full oral examination at a public dental care clinic within a year after their VIP screening and (ii) having answered at least 50 % of each of the SF-36 questions. Of these subjects, 9,838 had a second full caries examination 2–7 years (mean 95 % CI), 3.70 (3.67–3.72) years) after the baseline recording.

This study was approved by the Local Ethical Committee in Umeå, Sweden. All participants signed a written consent form at the health screening to participate and to allow that all information could be used for scientific purposes provided the results were published in an untraceable fashion.

### SF-36

The overall structure of the SF-36 instrument, including its eight dimensions, is presented in Additional file 1: Figure S1. The dimensions are physical functioning (PF), role physical (RP), bodily pain (BP), general health (GH), vitality (VT), social functioning (SF), role emotional (RE) and mental health (MH). The score of each scale falls between 0 and 100 (the higher the score, the better the HRQoL). The scales are combined in the Physical Component Summary score (PCS; weighted sum of PF, RP, BP and GH) and the Mental Component Summary score (MCS; weighted sum of VT, SF, RE and MH) [18]. In the present study, the proportion of missing answers for the SF-36 questions were well below 1 % for all but one question, for which it was 1.1 %. Hence, scores were computed for all respondents and missing values were replaced with Swedish reference means [13]. Similar to most previous studies, the questions on self-reported health transition were not evaluated [15, 21].

### Dental data

Information on the subjects’ number of teeth and ‘decayed, missing and filled’ tooth surfaces (DMFS) was retrieved from electronic records at the Public Dental Service of the County of Västerbotten. This was recorded by the participants’ usual dentist, and there was no calibration among the dental examiners. The caries examinations included a visual examination and at least two bitewing radiographs, and they were performed in clinics with state-of-the-art equipment. Data from the same year as the VIP visit and subsequent years were retrieved.

### Recording of potential medical and lifestyle confounders

Heights and body weights were measured when the person wore light clothes but no shoes. Blood samples were drawn and cardiovascular risk factors, i.e., total cholesterol levels, triglyceride levels, systolic and diastolic blood pressures, and fasting and 2-h blood sugar levels after a 75-g load of glucose in solution, were measured as previously described [19].

Information on education (primary or lower secondary education, upper secondary education and university), working conditions (including working night shift), any sick leave lasting ≥6 months and tobacco use (smoking and use of Swedish snuff, i.e., snus) was obtained by the questionnaire. Participants were classified as follows: (*i*) smokers if they were current daily or occasional smokers; (*ii*) ex-smokers if they had previously been a daily or occasional smoker; (*iii*) non-smokers if they had never smoked. Snuff use was classified as present, past or never used.

Dietary intake, including the intake of beer, wine and strong liquor, was recorded with a validated 66 item Food Frequency Questionnaire (FFQ) [22]. The reported intake frequencies were transformed into daily intakes of nutrients and servings of foods/food aggregates or grams of alcohol per day, as previously described [23]. FFQs in which the answers to ≥10 % of the questions were missing and those that yielded extreme estimated energy intake (lowest and highest 1 %) on the basis of the estimated food intake level (FIL = total energy intake/basal metabolic rate) [24] were excluded from the study. After these exclusions, 14,973 subjects were eligible for the evaluation of dietary intake.

Physical activity was estimated using the Cambridge Physical Activity Index [25] from self-reported activity at work and during leisure time.

### Data management and statistical analyses

The data were organised and analysed using the IBM Statistical Package for Social Sciences (SPSS, version 22.0). The primary outcome variables were DMFS (Decayed + Missing + Filled tooth surfaces, i.e., lifelong caries experience) and DMFS increment (follow-up DMFS - DMFS at baseline). The main exposure was SF-36 scores. The participants were classified into quintile groups based on their DMFS or SF-36 scores with ranking performed separately for men and women and for the 10-year age groups. The DMFS scores were normally distributed, whereas the SF-36 scores were right-skewed. For DMFS and for other normally distributed variables, the adjusted means (with 95 % confidence intervals (CIs)) are presented. For these data, differences between groups were tested by comparing GLM least square means standardised for sex, age, screening year and number of years between the VIP screening and dental recordings by parametric tests as described in the footnotes of the tables. For the SF-36 dimensions medians with percentile values are presented, but the mean values are also given to allow for comparisons with normative reference means [13, 15, 18]. Group differences were tested using the non-parametric Kruskal–Wallis tests. Differences in subject distributions were tested using the Chi-squared test.

Partial least squares (PLS) modelling was used to screen for factors potentially associated with DMFS at baseline (the dependent variable as a continuous measure) and subject characteristics as the set of independent variables. The software SIMCA P+ (v. 12.0; Umetrics AB, Umeå, Sweden) was used for PLS modelling. All variables were autoscaled to unit variance before being entered into the model. The importance of each independent variable in explaining the variation among the outcome variable (DMFS) was given in a PLS loading column plot with PLS correlation coefficients and 95 % CIs. PLS correlation coefficients where the 95 % CI did not include 0 were considered statistically significant. The R^2^ and Q^2^ values provided the capacity of the x-variables to explain (R^2^) and predict (Q^2^) the variance of the y-values (i.e., DMFS). Q^2^ values were obtained by cross-validation in which every seventh observation was left out of the model and predicted by a model from the remaining observations. This was repeated until all observations had been left out of the model once.

Based on the outcome in the PLS screening (i.e., identified potential confounders between caries status and HRQoL), the adjusted means or proportions were compared for the DMFS, PCS and MCS quintile groups, and linear trends and effect sizes were calculated, and potential interactions were tested by including the interaction term in GLM models.

Logistic regression, was used to calculate the odds ratios (with 95 % CI) for being in the lowest versus highest quintile of PCS scores by quintile group classification based on the DMFS distribution. Crude odds ratios and odds ratios adjusted for potential confounders were identified by trend analyses. *P*-values <0.05 were considered statistically significant and all tests were two-sided.

## Results

The distributions of the different SF-36 dimensions are presented in Table 1. It can be seen that the mean values are in accordance with published normative mean values for Swedish men and women. A general description of the study group, including the primary outcomes (DMFS and DMFS increment) and various potential confounders, is presented in Table 2. Adjusted mean DMFS values at baseline varied from 25 surfaces in 30-year-olds to 83 surfaces in 60-year-olds, and the mean incidence varied from 1.1 to 1.9 surfaces.

**Table 1:** SF-36 score mean and distribution based on 15,615 subjects (7,669 men and 7,946 women)

	Normative values^a ^		Percentile value						
		Mean	5 %	10 %	25 %	Median	75 %	90 %	95 %
Men									
Physical Component Summary (PCS)	50.6	50.5	31.9	38.2	47.9	53.0	56.1	57.6	58.3
Physical functioning (PF)	90	92	60	75	90	95	100	100	100
Role physical (RP)	85	87	0	50	100	100	100	100	100
Bodily pain (BP)	77	75	31	41	52	84	100	100	100
General health (GH)	77	76	40	50	67	77	90	97	100
Mental Component Summary (MCS)	50.0	51.9	34.8	42.2	49.9	54.2	56.6	58.6	59.3
Vitality (VT)	71	69	30	40	55	75	85	95	100
Social functioning (SF)	90	93	63	75	88	100	100	100	100
Role emotional (RE)	87	92	33	67	100	100	100	100	100
Mental health (MH)	82	85	56	64	80	88	92	100	100
Women									
Physical Component Summary (PCS)	49.5	48.5	27.9	33.4	43.6	51.4	55.8	57.8	58.7
Physical functioning (PF)	86	88	50	65	80	95	100	100	100
Role physical (RP)	82	82	0	25	75	100	100	100	100
Bodily pain (BP)	73	69	22	32	44	72	100	100	100
General health (GH)	75	73	35	40	60	77	88	97	100
Mental Component Summary (MCS)	49.1	50.1	28.8	36.1	47.2	53.2	56.1	58.4	59.6
Vitality (VT)	67	63	20	30	50	65	80	90	92
Social functioning (SF)	88	88	50	63	75	100	100	100	100
Role emotional (RE)	84	88	0	33	100	100	100	100	100
Mental health (MH)	80	81	52	60	72	84	92	96	100

^a^From normative data in [13, 15, 18]

**Table 2:** Study group characteristics of *n* = 15,615 study participants of whom 9,838 had a follow-up dental examination

	Age group (*n* = baseline/follow-up)				*p*-value	
	30-34 year (*n* = 161/108)	35-44 year (*n* = 5,664/3,579)	45-54 year (*n* = 5,261/3,290)	55-62 year (*n* = 4,529/2,861)	by age	by sex
Percent men/women	45 / 55	49 / 51	50 / 50	48 / 52		
Number of teeth^a^	29.4 (28.9-29.9)	28.9 (28.8-29.0)	27.7 (27.7-27.8)	24.7 (24.6-24.8)	<0.001	<0.001
Caries follow-up^b^, years	3.94 (3.68-4.23)	3.71 (3.66-3.76)	3.70 (3.65-3.75)	3.67 (3.62-3.72)	0.177	0.035
Caries status						
ᅟDMFT^a^	9.6 (8.8-10.4)	14.4 (14.3-14.6)	20.5 (20.4-20.6)	24.4 (24.2-24.5)	<0.001	0.321
ᅟDMFS^a^	24.9 (22.7-29.3)	37.2 (36.7-37.8)	58.6 (58.0-59.1)	82.8 (82.2-83.4)	<0.001	0.154
ᅟDMFS incidence^c^	1.1 (0.7-1.8)	1.1 (1.0-1.3)	1.5 (1.4-1.6)	1.9 (1.7-2.0)	<0.001	0.058
BMI^a^	26.0 (25.3-26.6)	26.1 (26.0-26.3)	26.6 (26.4-26.7)	26.9 (26.8-27.0)	<0.001	<0.001
Diet^a^						
ᅟenergy, kCal/day	1.907 (1.827-1.988)	1.756 (1.743-1.769)	1.724 (1.711-1.738)	1.661 (1.646-1.677)	<0.001	<0.001
ᅟcarbohydrates, E%	46.0 (44.9-47.0)	45.9 (45.8-46.1)	46.8 (46.6-47.0)	49.7 (49.5-49.9)	<0.001	<0.001
ᅟfat, E%	38.0 (36.9-39.0)	36.1 (35.9-36.3)	35.6 (35.4-35.7)	33.1 (32.9-33.3)	<0.001	<0.001
protein, E%	14.6 (14.2-15.0)	15.0 (14.9-15.1)	14.9 (14.8-14.9)	15.1 (15.0-15.2)	0.337	<0.001
sugar, E%	6.3 (5.8-6.7)	5.6 (5.5-5.7)	5.3 (5.3-5.4)	5.9 (5.8-6.0)	0.001	0.083
Alcohol^a^, g/day	3.6 (2.8-4.3)	3.8 (3.6-3.9)	4.1 (4.0-4.3)	4.2 (4.0-4.3)	0.717	<0.001
Smoking					<0.001	<0.001
ᅟpresent, %	15.6	11.5	17.3	15.9		
ᅟpast, %	20.6	20.8	33.9	41.1		
ᅟnever, %	63.7	67.7	48.9	43.0		
Snuff use					<0.001	<0.001
ᅟpresent, %	28.7	23.3	20.0	12.6		
ᅟpast, %	9.4	12.5	13.0	11.6		
ᅟnever, %	61.9	64.2	67.0	75.8		
University education, %	25.0	30.5	27.1	21.2	<0.001	<0.001
Physically inactive, %	12.5	14.5	15,4	18.0	<0.001	<0.001
Two or more medicines, %	0.6	1.3	5.0	15.5	<0.001	0.641
Sick leave ≥6 months, %	7.0	17.3	22.5	32.4	<0.001	<0.001

*N*-values are for numbers at baseline / numbers at follow-up

^a^Mean (95 % CI) adjusted for sex, age and screening year; ^b^Mean (95 % CI); ^c^mean (95 % CI) adjusted for sex, age, screening and follow-up years

As a first step, we compared the mean (95 % CI) DMFS increments and DMFS values at baseline in quintiles for the PCS and MCS scores and the eight SF-36 dimensions in men and women separately (Additional file 2: Table S1 and Table S2). In women, statistically significant trends were observed between increasing mean DMFS increments and decreasing RP, GH, MCS, VT, SF, RE, and MH scores (Additional file 2: Table S1), whereas no such associations were found in men (Additional file 2: Table S2). However, when lifelong experience of caries (DMFS) was used as the outcome, increasing DMFS values were associated with decreasing PCS scores, but not MCS scores, for both women and men (Additional file 2: Table S1 and Table S2). The mean DMFS values also increased with decreasing quintiles for each of the dimensions underlying the PCS score (i.e., PF, RP, BP and GH) in both men and women (Additional file 2: Table S1 and Table S2). For the dimensions underlying the MCS score, the mean DMFS values increased in women with decreasing quintile groups for all four dimensions (VT, SF, RE and MH) (Additional file 2: Table S1), but in men, a similar trend was observed for VT only (Additional file 2: Table S2).

**Fig. 1:**
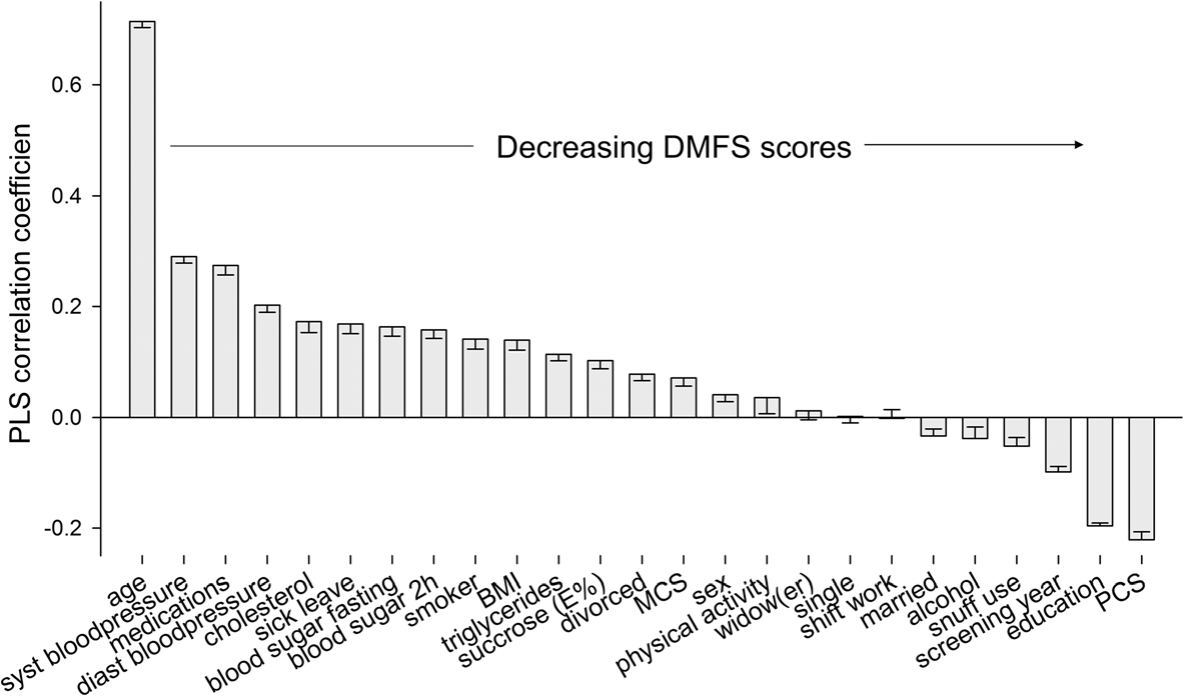
Column loading plot from PLS modelling of DMF surfaces at baseline. DMFS (continuous measure) was employed as the dependent variable and factors potentially associated with the risk of developing caries were the independent variables. The strength and directions of the associations are shown as PLS correlation coefficients on the y-axis. Factors with 95 % CIs that do not include zero are statistically significant. Those with PLS coefficients >0 are associated with more caries (to the left; i.e., the higher the age, the higher the caries score) and those with negative coefficients are associated with fewer caries (to the right; i.e., the higher the PCS score, the lower the caries score)

As a second step, we performed PLS multivariate modelling with caries prevalence as the dependent variable and factors reflecting lifestyle, physical aspects, and mental aspects as the independent block to identify variables associated with caries status that may have confounded the HRQoL. The PLS model revealed that higher caries scores were associated with age, systolic and diastolic blood pressure, number of medications, having taken ≥6 months of sick leave, cholesterol, triglyceride and blood sugar levels, BMI, smoking, sugar intake, and MCS score, being divorced and being physically inactive, and sex (Fig. 1). Lower caries scores were associated with higher PCS scores, higher education, screening year, snuff use, reported alcohol intake and being married (Fig. 1). The pattern was similar when analysed separately for men and women (data not shown). The model had an explanatory capacity of 46.0 % and a cross-validated predictive capacity of 45.8 % (R^2^ and Q^2^ values, respectively) for the two strongest components. Of these identified factors, only being divorced was found to have an interaction with the PCS and MCS scores.

PLS identifies hidden structures in the data swarm and clusters influential variables, but without any standardisations. Therefore, we followed-up on each of the factors identified in the screening PLS loading plot in quintile groups of caries prevalence, PCS scores and MCS scores with adjustments for sex, age, screening year and number of follow-up years (Additional file 2: Table S3 Table S4 and Table S5). The most influential factors for caries development were education level and smoking status, each with effect sizes of 15 and 12 %, respectively; in comparison, being single or married, using snuff, being physically inactive, number of medications, and having taken ≥6 months of sick leave had effect sizes between 3 % and 7 %, whereas sugar intake had an effect size of 0.6 % (Additional file 2: Table S3). These trends were similar in the sex and age strata (data not shown). Notably, standardisation for sex, age and screening year, eliminated the association between blood cholesterol, 2 h blood sugar, being divorced, snuff use and DMFS scores as indicated in the PLS loading plot.

The proportion of subjects with university education similarly increased with increasing quintile groups of PCS scores (effect size: 13 %; Additional file 2: Table S4); the opposite trend was observed for MCS scores (Additional file 2: Table S5). Furthermore, the lowest quintile of both PCS and MCS scores contained more single participants and fewer married participants (Additional file 2: Table S4 and Table S5). Several lifestyle markers, including smoking, snuff use, physical inactivity and sugar intake, were associated with decreasing quintile groups for both PCS and MCS scores (Additional file 2: Table S4 and Table S5). The cardio-metabolic risk factors, BMI, triglyceride and blood sugar levels and blood pressures increased with decreasing quintiles of PCS score (Additional file 2: Table S4). For MCS scores, similar trends were observed only for the triglyceride land fasting blood sugar levels, and blood pressure (Additional file 2: Table S5). The trends were similar for sex and age strata (data not shown). The number of medications increased with decreasing quintile groups of PCS and MCS scores, and the same trend was observed for sick leave (Additional file 2: Table S4 and Table S5).

Finally, we calculated the odds ratio for being in caries quintile 1-5 when having the poorest HRQOL (lowest PCS quintile) compared with the best HRQoL (highest PCS quintile) (Table 3). The crude odds ratio (β-coefficient with 95 % CI) for being in the highest versus in the lowest caries quintile at follow-up when being the lowest PCS quintile compared with the highest PCS quintile was 1.88 (1.54–2.31). The pattern was similar for men and women (data not shown). The odds ratio decreased to 1.57 (1.23–2.00) when the model was adjusted for education, smoking, having taken ≥6 months of sick leave and using two or more medications. Additional adjustment had no further effect. Restricting the analysis to the 9,838 subjects who had a follow-up yielded the same trend for the odds ratios as those presented in Table 3.

**Table 3:** Odds ratio (β-coefficient with 95 % confidence intervals (95 % CI)) if in the lowest (poorest) versus the highest (best) quintile of physical health quality of life (PCS scores)

Factor in model	β-coefficient	95 % CI	*p*-value
Crude model			
Caries			
ᅟQ1(lowest DMFS)	1.00		
ᅟQ2	1.07	0.879 - 1.31	0.490
ᅟQ3	1.18	0.97 - 1.44	0.107
ᅟQ4	1.52	1.25 - 1.85	<0.001
ᅟQ5 (highest DMFS)	1.88	1.54 - 2.31	<0.001
Adjusted model^a^			
Caries			
ᅟQ1(lowest DMFS)	1.00		
ᅟQ2	1.05	0.83 - 1.33	0.701
ᅟQ3	1.08	0.85 - 1.38	0.520
ᅟQ4	1.26	1.00 - 1.60	0.054
ᅟQ5 (highest DMFS)	1.57	1.23- 2.00	0.001
Education (no university)	1.82	1.53 - 2.17	<0.001
Smoking (present)	1.08	0.87- 1.34	0.478
Sick leave ≥6 months	10.80	9.00 - 12.95	<0.001
≥2 medications	3.61	3.40 - 5.43	<0.001

Ranking into caries quintile groups was for caries prevalence at follow-up by sex and 10-year age groups. Hence, age and sex was not included in the adjusted logistic regression analysis model

^a^adjustment for marital status, additional lifestyle measures, and medical measures had no further effect (cf. Additional file 2: Table S3)

## Discussion

In the present study, increasing caries incidence was associated with decreasing scores for RP, GH, VT, SF, RE, MH and MCS scores in women but not in men. However, the prevalence of caries (lifetime experience of caries) increased with decreasing PCS scores and each of the underlying dimensions in both men and women and with decreasing scores for the dimensions underlying the MCS score (VT, SF, RE and MH) in women. These associations likely represent confounding by, for example, education level, the number of medications prescribed, smoking status, and ever having taken at least 6 months sick leave. Subjects with a high prevalence of caries and low physical HRQoL were also characterised by marital status, snuff use, higher sugar intake, physical inactivity and having unfavourable levels of several lifestyle-related cardiovascular risk factors.

The strengths of the present study were that the study group represented the population in the entire County of Västerbotten in Northern Sweden (15,093 km^2^ in area, with approximately 262,000 inhabitants) and was large enough to allow for gender-separated analyses. A thoroughly validated instrument with population-based reference values was used to estimate HRQoL [13–16, 18] and the response rate was very high for all 36 SF-questions. Moreover, information on lifestyle habits and medical and socioeconomic status was collected by standardised routines at the same screening as the SF-36 [26]. Finally, clinically determined, as opposed to self-reported, caries status could be retrieved at baseline and at follow-up. Nonetheless, the study has limitations that should be considered. The participants were those who received dental care at the Public Dental Service in the County of Västerbotten, Sweden. The Public Dental Service provides regular dental care to approximately 45 % of the adult residents in this region [27]; most patients attend the same dental clinic on a long-term basis, with recall visits every 1–3 years. The remaining adult residents in the region either receive their dental care in private practices or do not seek regular dental care. The proportion that receives their dental care at the Public Dental Service is significantly higher in the northern-most counties compared with those in southern Sweden [27]. This pattern has arisen partly because of tradition and partly because the distance to a private clinic is often prohibitively large, whereas Public Dental Service is available at a clinic in the nearest community. The results from previous screenings do not indicate any major differences in dental or socioeconomic status among adults treated by the Public Dental Service or in private clinics in the Västerbotten region [20, 28]. Furthermore, the SF-36 scores were in accordance with the normative values from Sweden and other Western countries [13, 15, 18], supporting both the usefulness of the instrument in the Northern Sweden population and the representativeness of the study group. Taken together, we consider it unlikely that there was a severe systematic selection bias in the recruitment of participants in our study. Caries data were retrieved from electronic records at the Public Dental Service from dental examinations conducted by the participants’ regular dental care provider. The large number of dentists involved is likely to have balanced the risk of systematic under- or over-recordings. However, it is plausible that the proportion of residents with the poorest HRQoL is underrepresented in our study because only those who could visit a dental clinic are represented.

DMFS was calculated according to WHO definitions [28] and was used as the measure of dental caries. This index, which represents the sum of untreated caries lesions (restricted to cavitated lesions), fillings and other restorations, and missing teeth, aims at capturing caries experience over a lifetime. By this definition, the DMFS scores may be systematically underestimated if incipient caries are prevalent and overestimated if treatment guidelines favour operative over preventive treatments. In Sweden, the overall caries incidence is low [29], and minimally invasive approaches for caries treatment [30] have been applied for decades. This likely means that the F component is not generally over-estimated in this population. Furthermore, under-estimations from the exclusion of white spot lesions is likely more pronounced in younger age groups compared to middle age groups with more stable caries, such as the group used in this study. The M component, which should include tooth losses due to caries, is biased by losses due to orthodontic treatment and periodontal disease. However, information on why a tooth was lost is, in general, unavailable. The WHO rule is to include all tooth losses.

The present study evaluated whether subjects reporting a poor HRQoL were more likely to develop or have more caries than those with better scores, and what the linking or confounding aspects might be. Such aspects are likely to involve both underlying medical aspects and psycho-social aspects that impact coping with caries-inducing factors, such as diet and oral hygiene. Hence, the aspects linking poor HRQoL to caries development may involve eating habits, illness, handicap, insomnia, and polypharmacy with impaired saliva secretion and cognitive capacity, which may be reflected in the individuals’ education levels, smoking statuses, BMI, and cardio-metabolic risk factors. In line with this, education level, markers for medical issues (e.g., sick leave, and medication), and lifestyle were found to be associated with both HRQoL and lifetime experience of caries in both men and women; for women, these factors were also associated with prospective caries development. These findings are in line with those reported by Costa and co-workers in Brazilian adults [31]. Both our findings and the results of Costa and co-workers are, however, in contrast with findings in American adolescents [11]. In general, it is difficult, if not impossible, to distinguish between causal and confounding associations in descriptive epidemiological studies involving complex diseases, such as caries, type-2 diabetes and obesity [5, 32]. The determinants for such conditions are multifactorial, with different mosaics for subgroups of subjects as shown for early childhood caries [33]. Among the identified factors shared by HRQoL and caries, most did not represent a potential causal association, but were instead confounding factors. One example of a newer epidemiological technique that has the capacity to circumventing confounding factors and identifying causal associations is the Mendelian randomisation method, in which genes associated with the outcome or exposure are employed. We recently applied this method to show that the commonly reported association between obesity and periodontal disease is unlikely to be causal [34].

In the present study, decreasing SF-36 scores were associated with increasing caries incidence in women, whereas such associations were found with the lifelong, cumulative experience of caries in both men and women. This may reflect that the association between HRQoL and caries incidence in middle-aged people is limited to women. Alternatively, it may reflect that the power to detect an association was significantly lower for caries increments due to less variation in caries incidences compared to the lifelong, cumulative experience of caries. In populations with organised dental care, such as in Sweden, caries is a condition that progresses slowly, with symptoms taking on average 4–5 years to become clinically detectable even with the use of excellent light and x-rays [35, 36]. In the present study, the follow-up time was mostly 2-4 years. Hence, the follow-up period may have been too short to properly evaluate caries incidence.

Evaluating the effect size provided an estimate of the magnitude of the association between caries and SF-36 scores and with each of the identified confounding/causal factors. For several of the SF-36 dimensions, decreasing SF-36 scores were associated with a linear increase in caries, but the effect sizes were moderate. Moreover, the average difference between the highest and lowest SF-36 quintile was five DMF surfaces, which is equivalent to the loss of one tooth. Nevertheless, the odds ratio for having the poorest caries status increased by 88 % (57 % if adjustments were performed) if a subject had the poorest rather than the best physical HRQoL.

## Conclusion

Based on the present findings, we conclude that some dimensions of mental HRQoL were associated with higher caries incidence in women, but both physical aspects of HRQoL and some mental dimensions were associated with the lifelong experience of caries in both men and women. The mechanisms underlying the identified associations, which likely involve confounding biological and lifestyle factors as well as living conditions, remain to be elucidated. The study implies that, when possible, subjects with poor HRQoL would benefit from caries prevention measures meeting the underlying situation. Such measures would rely on a functioning collaboration with medical disciplines and cost-effective monitoring.

## Additional files

Additional file 1: Figure S1. Schematic description of the aspects monitored by the SF-36 instrument as measures of health-related quality of life. (PPTX 98 kb) Additional file 2: Table S1. DMFS and DMFS increase from baseline to follow-up in women in SF-36 quintile strata. The data are shown as the means (95 % CIs) adjusted for age, screening year, and years to follow-up. Numbers in the strata vary by distribution of scores for each SF-36 dimension; strata with <200 observations were not considered. **Table S2.** DMFS and DMFS increase from baseline to follow-up in men in SF-36 quintile strata. The data are shown as the means (with 95 % CIs) adjusted for age, screening year, and years to follow-up as indicated in the footnote. Numbers in the strata vary by distribution of scores for each SF-36 dimension; strata with <200 observations were not considered. **Table S3.** Social situation, lifestyle and medical characteristics in all participants by quintile classification of caries prevalence at follow-up. **Table S4.** Social situation, lifestyle and medical characteristics in all participants by quintile classification of Physical Component Summary scores (PCS). **Table S5.** Social situation, lifestyle and medical characteristics in all participants by quintile classification of Mental Component Summary scores (MCS). (DOCX 67 kb)

## Abbreviations

BMI: body mass index
BP: bodily pain
CI: confidence interval
DMFS: decayed, missing and filled tooth surfaces
DMFT: Decayed, missing and filled teeth
E%: energy percentage
FFQ: food frequency questionnaire
FIL: food intake level(total energy intake/basal metabolic rate)
GH: general health perceptions
HRQoL: health-related quality of life
kCal: kilocalorie
MCS: mental component summary score
MH: mental health
OIDP: the oral impacts on daily performance
PCS: physical component summary score
PF: physical functioning
PLS: partial least squares regression analysis
RE: role emotional
RP: role physical
R^2^: symbol for the coefficient of determination of a linear regression
SF: social functioning
SF-36: the 36-question short form instrument
SPSS: IBM statistical package for social sciences
QOL: quality of life
Q^2^: estimates goodness of prediction in PLS
VIP: Västerbotten intervention programme
VT: vitality
WHO: the World Health Organization
