# Supplementary material for: Health-related quality of life and prospective caries development
Source: BMC Oral Health. 2016 Feb 9;16:15. doi: 10.1186/s12903-016-0166-3 (PMC4746799; doi:10.1186/s12903-016-0166-3)
Supplement: Additional file 1: Figure S1. — Schematic description of the aspects monitored by the SF-36 instrument as measures of health-related quality of life. (PPTX 98 kb) [file 12903_2016_166_MOESM1_ESM.pptx]

## Slide 1
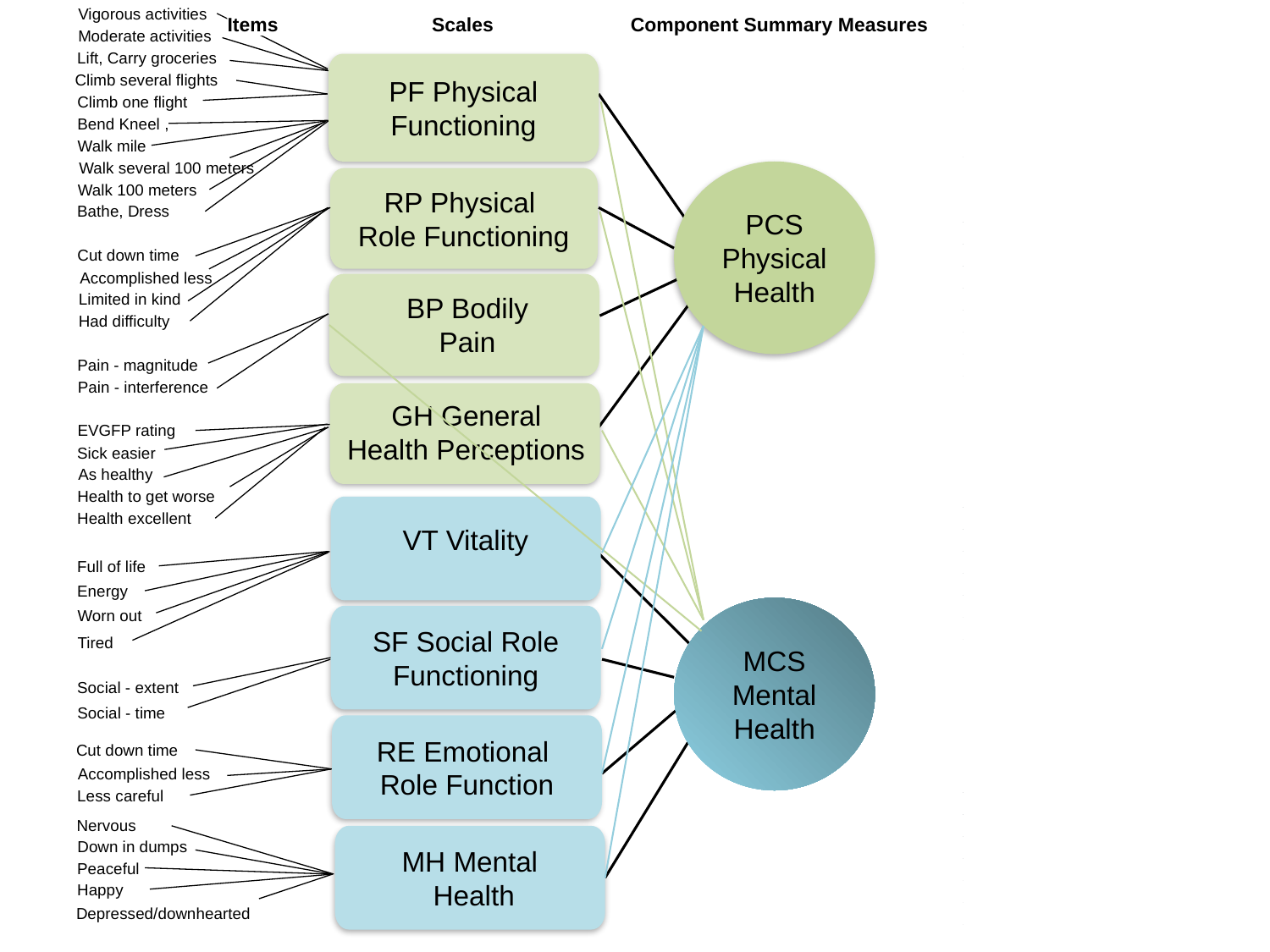

Vigorous activities
Moderate activities
Lift, Carry groceries
Climb several flights
Climb one flight
Bend Kneel ,
Walk mile
Walk several 100 meters
RP Physical
Role Functioning
Walk 100 meters
Bathe, Dress
Cut down time
Accomplished less
Limited in kind
Had difficulty
Pain - magnitude
Pain - interference
EVGFP rating
Sick easier
As healthy
Health to get worse
Health excellent
Full of life
Energy
Worn out
Tired
Social - extent
Social - time
Cut down time
Accomplished less
Nervous
Down in dumps
Peaceful
Happy
Depressed/downhearted
Items Scales Component Summary Measures
PF Physical Functioning
PCS
Physical Health
BP Bodily
Pain
GH General
Health Perceptions
VT Vitality
MCS
Mental Health
SF Social Role
Functioning
RE Emotional
Role Function
Less careful
MH Mental
 Health
