# Supplementary material for: Health-related quality of life and prospective caries development
Source: BMC Oral Health. 2016 Feb 9;16:15. doi: 10.1186/s12903-016-0166-3 (PMC4746799; doi:10.1186/s12903-016-0166-3)
Supplement: Additional file 2: Table S1. — DMFS and DMFS increase from baseline to follow-up in women in SF-36 quintile strata. The data are shown as the means (95 % CIs) adjusted for age, screening year, and years to follow-up. Numbers in the strata vary by distribution of scores for each SF-36 dimension; strata with <200 observations were not considered. Table S2. DMFS and DMFS increase from baseline to follow-up in men in SF-36 quintile strata. The data are shown as the means (with 95 % CIs) adjusted for age, screening year, and years to follow-up as indicated in the footnote. Numbers in the strata vary by distribution of scores for each SF-36 dimension; strata with <200 observations were not considered. Table S3. Social situation, lifestyle and medical characteristics in all participants by quintile classification of caries prevalence at follow-up. Table S4. Social situation, lifestyle and medical characteristics in all participants by quintile classification of Physical Component Summary scores (PCS). Table S5. Social situation, lifestyle and medical characteristics in all participants by quintile classification of Mental Component Summary scores (MCS). (DOCX 67 kb) [file 12903_2016_166_MOESM2_ESM.docx]

**Additional table 1. DMFS and DMFS increase from baseline to follow-up in women in SF-36 quintile strata.** The data are shown as the means (95% CIs) adjusted for age, screening year, and years to follow-up. Numbers in the strata vary by distribution of scores for each SF-36 dimension; strata with <200 observations were not considered.

|  | SF-36 quintile group | | | | |  | P-value for  trend | Effect  Size |
| --- | --- | --- | --- | --- | --- | --- | --- | --- |
| **WOMEN** |  |  |  |  |  |  |  |  |
|  | 1^st^ quintile | 2^nd^ quintile | 3^rd^ quintile | 4^th^ quintile | 5^th^ quintile |  |  |  |
| Numbers | 803-1,025 | 518-1,119 | 877-1,160 | 506-3,479 | 816-1,439 |  |  |  |
| **DMFS at baseline^1^** |  |  |  |  |  |  |  |  |
| Physical Component Summary (PCS) | 64.2 (62.9-65.5) | 63.5 (62.3-64.8) | 61.3 (60.1-62.6) | 59.8 (58.5-61.0) | 59.2 (57.8-60.5) |  | <0.001 | 0.009 |
| physical function (PF) | 64.6 (63.3-65.8) | 62.8 (61.4-64.2) | 60.9 (59.6-62.1) | 60.8 (59.6-62.0) | 58.9 (57.4-60.3) |  | <0.001 | 0.008 |
| role physical (RP) | 64.0 (62.7-65.3) | 62.0 (60.2-63.8) | - | 61.4 (60.7-62.1) | − |  | 0.001 | 0.002 |
| bodily pain (BP) | 65.2 (63.9-66.6) | 62.9 (61.7-64.1) | 61.1 (59.8-62.4) | 59.4 (57.6-61.3) | 59.4 (58.3-60.5) |  | <0.001 | 0.011 |
| general health (GH) | 65.0 (63.7-66.3) | 62.2 (60.9-63.5) | 61.8 (60.6-63.0) | 59.9 (58.6-61.2) | 58.9 (57.5-60.3) |  | <0.001 | 0.010 |
| Mental Component Summary (MCS) | 62.4 (61.1-63.7) | 62.3 (61.0-63.6) | 60.2 (58.9-61.5) | 61.5 (60.2-62.8) | 61.8 (60.5-63.0) |  | 0.290 | - |
| vitality (VT) | 63.2 (61.9-64.6) | 62.6 (61.3-63.8) | 62.0 (60.7-63.4) | 60.4 (59.2-61.7) | 60.0 (58.7-61.2) |  | <0.001 | 0.004 |
| social functioning (SF) | 63.4 (62.0-64.8) | 61.0 (59.8-62.3) | − | 61.4 (60.6-62.1) | − |  | 0.014 | 0.001 |
| role emotional (RE) | 63.4 (62.1-64.8) | − | 61.2 (60.6-61.9) | − | − |  | 0.003 | 0.002 |
| mental health (MH) | 63.4 (62.0-64.7) | 63.0 (61.6-64.3) | 60.1 (58.9-61.4) | 60.8 (59.7-62.0) | 61.3 (59.9-62.7) |  | 0.004 | 0.004 |
| **DMFS-incidence^2^** |  |  |  |  |  |  |  |  |
| Physical Component Summary (PCS) | 1.8 (1.6-2.1) | 1.7 (1.4-2.0) | 1.3 (1.0-1.6) | 1.3 (1.1-1.6) | 1. 7 (1.4-1.9) |  | 0.113 | - |
| physical function (PF) | 1.9 (1.6-2.2) | 1.7 (1.4-1.9) | 1.1 (0.9-1.4) | 1.6 (1.3-1.8) | 1.5 (1.2-1.8) |  | 0.066 | - |
| role physical (RP) | 2.0 (1.8-2.3) | 1.6 (1.2-1.9) | − | 1.4 (1.3-1.6) | − |  | <0.001 | 0.003 |
| bodily pain (BP) | 1.7 (1.5-2.0) | 1.6 (1.4-1. 9) | 1.4 (1.1-1.7) | 1.3 (0.9-1.7) | 1.6 (1.4-1.8) |  | 0.126 | - |
| general health (GH) | 2.0 (1.7-2.3) | 1.4 (1.1-1.7) | 1.4 (1.1-1.7) | 1.6 (1.4-1.8) | 1.4 (1.1-1.7) |  | 0.019 | 0.003 |
| Mental Component Summary (MCS) | 2.2 (2.0-2.5) | 1.7 (1.4-1.9) | 1.3 (1.0-1.6) | 1.3 (1.0-1.5) | 1.4 (1.1-1.6) |  | <0.001 | 0.007 |
| vitality (VT) | 2.1 (1.9-2.4) | 1.6 (1.3-1.8) | 1.5 (1.2-1.7) | 1.3 (1.0-1.6) | 1.4 (1.2-1.7) |  | <0.001 | 0.004 |
| social functioning (SF) | 2.0 (1.7-2.3) | 1.6 (1.4-1.9) | − | 1.4 (1.3-1.6) | − |  | 0.001 | 0.002 |
| role emotional (RE) | 2.2 (1.9-2.4) | − | 1.4 (1.3-1. 6) | − | − |  | <0.001 | 0.004 |
| mental health (MH) | 2.1 (1.8-2.4) | 1.7 (1.4-1.9) | 1.3 (1.1-1.6) | 1.4 (1.2-1.6) | 1.4 (1.1-1.7) |  | <0.001 | 0.004 |
| 1. Means (95% CI) are adjusted for age, and screening year. 2. Means (95% CI) are adjusted for age, screening year, and years to follow-up. | | | | | | | | |

**Additional table 2. DMFS and DMFS increase from baseline to follow-up in women in SF-36 quintile strata.** The data are shown as the means (95% CIs) adjusted for age, screening year, and years to follow-up. Numbers in the strata vary by distribution of scores for each SF-36 dimension; strata with <200 observations were not considered.

|  | SF-36 quintile group | | | | |  | P-value  for  trend | Effect  Size |
| --- | --- | --- | --- | --- | --- | --- | --- | --- |
| **MEN** | 1^st^ quintile | 2^nd^ quintile | 3^rd^ quintile | 4^th^ quintile | 5^th^ quintile |  |  |  |
| Numbers | 791-961 | 435-1,126 | 759-1,465 | 903-3,571 | 459-1,104 |  |  |  |
| **DMFS at follow-up^1^** |  |  |  |  |  |  |  |  |
| Physical Component Summary (PCS) | 62.0 (60.7-63.3) | 61.3 (60.0-62.7) | 58.2 (56.9-59.5) | 58.4 (57.1-59.7) | 57.1 (55.7-58.4) |  | <0.001 | 0.008 |
| physical function PF) | 62.3 (60.9-63.6) | 60.3 (58.9-61.7) | 58.5 (56.9-60.1) | 58.2 (57.2-59.3) | 57.9 (55.7-60.1) |  | <0.001 | 0.006 |
| role physical (RP) | 60.7 (59.3-62.0) | - | 59.0 (57.4-60.5) | 58.0 (56.9-59.1) | − |  | 0.003 | 0.002 |
| bodily pain (BP) | 62.1 (60.8-63.4) | 60.1 (58.7-61.5) | 58.5 (57.2-59.7) | 58.6 (56.8-60.3) | 57.8 (56.5-59.1) |  | <0.001 | 0.006 |
| general health (GH) | 62.0 (60.6-63.3) | 59.8 (58.6-61.1) | 59.1 (57.8-60.5) | 58.3 (57.0-59.6) | 57.7 (56.3-59.1) |  | <0.001 | 0.005 |
| Mental Component Summary (MCS) | 60.0 (58.7-61.4) | 58.6 (57.3-60.0) | 58.5 (57.2-59.8) | 59.5 (58.2-60.9) | 60.1 (58.8-61.4) |  | 0.618 | - |
| vitality (VT) | 60.7 (59.4-62.1) | 60.1 (58.7-61.5) | 59.4 (58.1-60.7) | 58.0 (56.6-59.4) | 58.8 (57.5-60.0) |  | 0.005 | 0.002 |
| social functioning (SF) | 60.8 (59.3-62.3) | 59.2 (57.2-61.2) | − | 59.5 (58.8-60.2) | − |  | 0.113 | - |
| role emotional (RE) | 59.7 (57.9-61.4) | − | 59.3 (58.7-60.0) | − | − |  | 0.742 | - |
| mental health (MH) | 60.9 (59.5-62.3) | 59.1 (57.9-60.4) | 57.8 (56.4-59.2) | 59.2 (58.0-60.5) | 59.9 (58.6-61.2) |  | 0.404 | - |
| **DMFS-incidence^2^** |  |  |  |  |  |  |  |  |
| Physical Component Summary (PCS) | 1.5 (1.3-1.8) | 1.4 (1.1-1.6) | 1.2 (1.0-1.5) | 1.4 (1.2-1.6) | 1.4 (1.2-1.6) |  | 0.421 | - |
| physical function (PF) | 1.5 (1.3-1.8) | 1.4 (1.2-1.7) | 1.4 (1.1-1.6) | 1.4 (1.2-1.5) | 1.2 (0.8-1.6) |  | 0.112 | - |
| role physical (RP) | 1.5 (1.3-1.7) | − | 1.3 (1.0-1.5) | 1.4 (1.2-1.6) | − |  | 0.545 | - |
| bodily pain (BP) | 1.6 (1.4-1.8) | 1.2 (1.0-1.5) | 1.31(1.1-1.5) | 1.3 (1.0-1.5) | 1.5 (1.3-1.7) |  | 0.629 | - |
| general health (GH) | 1.6 (1.3-1.8) | 1.4 (1.2-1.6) | 1.3 (1.1-1.5) | 1.4 (1.2-1.6) | 1.3 (1.1-1.4) |  | 0.171 | - |
| Mental Component Summary (MCS) | 1.6 (1.3-1.8) | 1.3 (1.1-1.6) | 1.2 (1.0-1.4) | 1.4 (1.1-1.6) | 1.5 (1.3-1.7) |  | 0.641 | - |
| vitality (VT) | 1.6 (1.4-1.9) | 1.4 (1.1-1.6) | 1.3 (1.1-1.5) | 1.3 (1.0-1.5) | 1.4 (1.2-1.6) |  | 0.121 | - |
| social functioning (SF) | 1.5 (1.2-1.7) | 1.3 (0.9-1.6) | − | 1.4 (1.3-1.5) | − |  | 0.420 | - |
| role emotional (RE) | 1.4 (1.1-1.7) | − | 1.4 (1.3-1.5) | − | − |  | 0.923 | - |
| mental health (MH) | 1.5 (1.2-1.7) | 1.4 (1.2-1.6) | 1.2 (1.0-1.5) | 1.3 (1.1-1.5) | 1.5 (1.2-1.7) |  | 0.752 | - |
| 1. Means (95% CI) are adjusted for age, and screening year. 2. Means (95% CI) are adjusted for age, screening year, and years to follow-up. | | | | | | | | |

**Additional table 3. Social situation, and life style and medical characteristics in all participants by quintile classification from caries prevalence distribution at follow-up.**

|  | Caries prevalence at follow-up quintile group | | | | |  | P-value for trend | Effect size |
| --- | --- | --- | --- | --- | --- | --- | --- | --- |
|  | 1^st^ quintile | 2^nd^ quintile | 3^rd^ quintile | 4^th^ quintile | 5^th^ quintile |  |  |  |
| **Social situation** |  |  |  |  |  |  |  |  |
| University education, % | 33.4 | 30.5 | 25.8 | 20.8 | 14.9 |  | <0.001 | 0.153 |
| Marital status |  |  |  |  |  |  |  |  |
| single, % | 7.8 | 8.1 | 8.5 | 9.0 | 12.6 |  | <0.001 | 0.059 |
| married, % | 83.7 | 83.4 | 83.5 | 81.5 | 79.0 |  | <0.001 | 0.0471 |
| divorced, % | 1.5 | 1.6 | 1.2 | 1.5 | 1.5 |  | 0.911 | - |
| widower, % | 7.0 | 6.9 | 6.7 | 8.0 | 7.0 |  | 0.540 | - |
| **Lifestyle** |  |  |  |  |  |  |  |  |
| Present smoker, % | 10.7 | 11.4 | 13.5 | 16.5 | 22.8 |  | <0.001 | 0.124 |
| Present snuff user, % | 16.9 | 18.9 | 18.6 | 21.3 | 22.7 |  | 0.001 | 0.052 |
| Alcohol^1^, g/day | 4.4 (4.2-4.6) | 4.3 (4.1-4.5) | 4.1 (3.8-4.3) | 3.8 (3.5-4.0) | 3.5 (3.3-3.8) |  | <0.001 | 0.003 |
| Sugar intake, E% | 5.6 (5.5-5.7) | 5.5 (5.4-5.6) | 5.6 (5.5-5.8) | 5.8 (5.7-5.9) | 6.1 (5.9-6.2) |  | <0.001 | 0.006 |
| Physical inactive, % | 15.1 | 15.4 | 13.7 | 16.6 | 16.9 |  | 0.048 | 0.031 |
| **Medical measures** |  |  |  |  |  |  |  |  |
| BMI^1^, kg/m^2^ | 25.7 (25.6-25.9) | 26.1 (26.0-26.3) | 26.3 (26.1-26.5) | 26.8 (26.6-27.0) | 27.2 (27.0-27.4) |  | <0.001 | 0.013 |
| Total cholesterol^1^, mmol/l | 5.33 (5.28-5.38) | 5.31 (5.26-5.35) | 5.29 (5.25-5.34) | 5.28 (5.23-5.32) | 5.28 (5.24-5.33) |  | 0.099 | - |
| Triglycerides^1^, mmol/l | 1.28 (1.25-1.32) | 1.34 (1.31-1.38) | 1.35 (1.31-1.38) | 1.40 (1.36-1.43) | 1.44 (1.40-1.47) |  | <0.001 | 0.004 |
| Blood sugar 0 h^1^, mmol/l | 5.42 (5.38-5.47) | 5.47 (5.43-5.51) | 5.48 (5.44-5.52) | 5.48 (5.44-5.52) | 5.54 (5.50-5.59) |  | <0.001 | 0.002 |
| Blood sugar 2 h^1^, mmol/l | 6.71 (6.64-6.78) | 6.76 (6.69-6.82) | 6.76 (6.69-6.83) | 6.82 (6.75-6.89) | 6.73 (6.66-6.80) |  | 0.321 | - |
| Systolic blood pressure^1^, mmHg | 124.1 (123.4-124.8) | 125.3 (124.6-126.0) | 125.3 (124.6-126.0) | 125.7 (125.0-126.4) | 126.4 (125.7-127.1) |  | <0.001 | 0.002 |
| Diastolic blood pressure^1^, mmHg | 77.0 (76.5-77.4) | 77.6 (77.2-78.1) | 77.6 (77.1-78.0) | 78.1 (77.6-78.5) | 78.3 (77.8-78.7) |  | <0.001 | 0.002 |
| Two or more medicines, % | 4.9 | 5.7 | 5.6 | 7.2 | 7.6 |  | 0.001 | 0.042 |
| Sick leave ≥6 months, % | 19.1 | 21.5 | 22.5 | 24.8 | 27.9 |  | <0.001 | 0.071 |

1) Mean values are standardized for sex, age, screening year, and years to follow-up.

**Additional table 4. Social situation, and life style and medical characteristics in all participants by quintile classification from the Physical Component Summary (PCS) distribution.**

|  | Physical Component Summary (PCS) quintile group | | | | |  | P-value for trend | Effect size |
| --- | --- | --- | --- | --- | --- | --- | --- | --- |
|  | 1^st^ quintile | 2^nd^ quintile | 3^rd^ quintile | 4^th^ quintile | 5^th^ quintile |  |  |  |
| **Social situation** |  |  |  |  |  |  |  |  |
| University education, % | 19.5 | 21.4 | 27.9 | 29.6 | 34.7 |  | <0.001 | 0.126 |
| Marital status |  |  |  |  |  |  |  |  |
| single, % | 11.7 | 9.9 | 9.2 | 8.8 | 9.0 |  | <0.001 | 0.037 |
| married, % | 78.4 | 81.4 | 81.5 | 82.4 | 81.4 |  | 0.002 | 0.035 |
| Divorced, % | 1.4 | 1.5 | 1.4 | 1.4 | 1.5 |  | 0.984 | - |
| widower, % | 8.4 | 7.1 | 7.9 | 7.4 | 8.2 |  | 0.893 | - |
| **Lifestyle** |  |  |  |  |  |  |  |  |
| Present smoker, % | 16.7 | 16.3 | 15.5 | 13.8 | 11.5 |  | <0.001 | 0.054 |
| Present snuff user, % | 21.1 | 20.5 | 19.0 | 18.8 | 16.6 |  | <0.001 | 0.040 |
| Alcohol^1^, g/day | 3.7 (3.5-3.8) | 3.8 (3.7-4.0) | 4.2 (4.0-4.3) | 4.1 (3.9-4.3) | 4.3 (4.2-4.5) |  | <0.001 | 0.002 |
| Sugar intake^1^, E% | 5.7 (5.6-5.8) | 5.6 (5.5-5.7) | 5.6 (5.5-5.7) | 5.5 (5.4-5.6) | 5.5 (5.4-5.6) |  | 0.001 | 0.001 |
| Physically inactive, % | 20.3 | 16.0 | 15.8 | 13.8 | 13.2 |  | <0.001 | 0.068 |
| **Medical measures** |  |  |  |  |  |  |  |  |
| BMI^1^, kg/m^2^ | 27.6 (27.5-27.8) | 27.1 (27.0-27.3) | 26.5 (26.4-26.7) | 26.0 (25.8-26.1) | 25.3 (25.1-25.4) |  | <0.001 | 0.036 |
| Total cholesterol^1^, mmol/l | 5.31 (5.28-5.35) | 5.29 (5.25-5.32) | 5.32 (5.28-5.35) | 5.30 (5.26-5.33) | 5.27 (5.23-5.31) |  | 0.231 | - |
| Triglycerides^1^, mmol/l | 1.50 (1.47-1.53) | 1.39 (1.36-1.42) | 1.36 (1.33-1.38) | 1.31 (1.28-1.33) | 1.21 (1.18-1.24) |  | <0.001 | 0.015 |
| Blood sugar 0 h^1^, mmol/l | 5.56 (5.53-5.60) | 5.52 (5.48-5.55) | 5.49 (5.46-5.53) | 5.44 (5.40-5.47) | 5.37 (5.34-5.41) |  | <0.001 | 0.005 |
| Blood sugar 2 h^1^, mmol/l | 6.88 (6.82-6.94) | 6.81 (6.76-6.87) | 6.76 (6.70-6.81) | 6.69 (6.63-6.74) | 6.61 (6.55-6.67) |  | <0.001 | 0.004 |
| Systolic blood pressure^1^, mmHg | 126.2 (125.7-126.7) | 126.1 (125.6-126.6) | 125.5 (125.0-126.0) | 124.8 (124.3-125.4) | 123.6 (123.1-124.2) |  | <0.001 | 0.004 |
| Diastolic blood pressure^1^, mmHg | 78.8 (78.4-79.1) | 78.5 (78.2-78.9) | 78.2 (77.8-78.5) | 77.7 (77.3-78.0) | 76.7 (76.3-77.0) |  | <0.001 | 0.006 |
| 2 or more medicines^1^, % | 12.5 | 8.9 | 5.6 | 4.1 | 2.2 |  | <0.001 | 0.146 |
| Sick leave ≥6 months^1^, % | 55.8 | 27.4 | 14.9 | 9.3 | 9.8 |  | <0.001 | 0.411 |

1. Mean values are standardized for sex, age, and screening year.

**Additional table 5. Social situation, and life style and medical characteristics in all participants by quintile classification from Mental Component Summary (MCS) distribution.**

|  | Mental Component Summary (MCS) quintile group | | | | |  |  | Effect size |
| --- | --- | --- | --- | --- | --- | --- | --- | --- |
|  | 1^st^ quintile | 2^nd^ quintile | 3^rd^ quintile | 4^th^ quintile | 5^th^ quintile |  | P-value for trend |  |
| **Social situation** |  |  |  |  |  |  |  |  |
| University education, % | 28.4 | 28.7 | 28.6 | 25.8 | 21.5 |  | <0.001 | 0.062 |
| Marital status |  |  |  |  |  |  |  |  |
| single, % | 12.2 | 10.5 | 8.9 | 8.1 | 8.8 |  | <0.001 | 0.049 |
| married, % | 74.4 | 79.8 | 82.2 | 84.6 | 84.3 |  | <0.001 | 0.096 |
| divorced, % | 1.8 | 1.6 | 1.4 | 1.1 | 1.3 |  | 0.018 | 0.021 |
| widower, % | 11.7 | 8.1 | 7.5 | 6.2 | 5.6 |  | <0.001 | 0.078 |
| **Lifestyle** |  |  |  |  |  |  |  |  |
| Present smoker, % | 18.2 | 14.5 | 14.1 | 14.0 | 13.0 |  | <0.001 | 0.050 |
| Present snuff user, % | 21.9 | 20.8 | 19.6 | 17.6 | 16.1 |  | <0.001 | 0.053 |
| Alcohol^1^, g/day | 4.1 (3.9-4.3) | 4.2 (4.0-4.5) | 4.1 (3.9-4.3) | 4.0 (3.8-4.1) | 3.7 (3.5-3.9) |  | <0.001 | 0.001 |
| Sugar intake^1^, E% | 5.8 (5.7-5.8) | 5.7 (5.6-5.8) | 5.6 (5.5-5.7) | 5.5 (5.4-5.6) | 5.4 (5.3-5.5) |  | <0.001 | 0.002 |
| Physically inactive, % | 20.9 | 16.6 | 15.2 | 12.2 | 14.1 |  | <0.001 | 0.079 |
| **Medical measures** |  |  |  |  |  |  |  |  |
| BMI^1^, kg/m^2^ | 26.8 (26.7-27.0) | 26.3 (26.2-26.5) | 26.2 (26.0-26.3) | 26.3 (26.1-26.4) | 27.0 (26.8-27.1) |  | 0.232 | - |
| Total cholesterol^1^, mmol/l | 5.28 (5.25-5.32) | 5.32 (5.28-5.35) | 5.29 (5.25-5.32) | 5.29 (5.25-5.33) | 5.31 (5.27-5.34) |  | 0.739 | - |
| Triglycerides^1^, mmol/l | 1.40 (1.37-1.42) | 1.36 (1.34-1.39) | 1.33 (1.30-1.36) | 1.32 (1.29-1.34) | 1.36 (1.33-1.38) |  | 0.005 | 0.001 |
| Blood sugar 0 h^1^, mmol/l | 5.52 (5.49-5.55) | 5.49 (5.46-5.53) | 5.45 (5.42-5.49) | 5.43 (5.40-5.46) | 5.49 (5.45-5.52) |  | 0.013 | 0.001 |
| Blood sugar 2 h^1^, mmol/l | 6.78 (6.73-6.84) | 6.73 (6.68-6.79) | 6.74 (6.68-6.79) | 6.71 (6.66-6.77) | 6.78 (6.72-6.83) |  | 0.761 | - |
| Systolic blood pressure^1^, mmHg | 124.6 (124.0-125.2) | 124.9 (124.3-125.4) | 124.9 (124.4-125.5) | 125.3 (124.8-125.9) | 126.5 (126.0-127.1) |  | <0.001 | 0.002 |
| Diastolic blood pressure^1^, mmHg | 77.7 (77.3-78.0) | 77.9 (77.5-78.2) | 77.8 (77.5-78.2) | 78.0 (77.6-78.3) | 78.5 (78.2-78.9) |  | 0.001 | 0.001 |
| 2 or more medicines, % | 10.5 | 6.4 | 5.2 | 5.1 | 6.1 |  | <0.001 | 0.079 |
| Sick leave ≥6 months, % | 38.0 | 22.0 | 17.3 | 15.3 | 23.8 |  | <0.001 | 0.189 |

1. Mean values are standardized for sex, age, and screening year.
